# Correction to ‘Genetic variation in genes regulating skeletal muscle regeneration and tissue remodelling associated with weight loss in chronic obstructive pulmonary disease’ by Lakshman Kumar et al.

**DOI:** 10.1002/jcsm.13442

**Published:** 2024-02-07

In Preeti Lakshman Kumar, Ava C. Wilson, Alison Rocco, Michael H. Cho, Emily Wan, Brian D. Hobbs, George R. Washko, Victor E. Ortega, Stephanie A. Christenson, Xingnan Li, J. Michael Wells, Surya P. Bhatt, Dawn L. DeMeo, Sharon M. Lutz, Harry Rossiter, Richard Casaburi, Stephen I. Rennard, David A. Lomas, Wassim W. Labaki, Ruth Tal‐Singer, Russel P. Bowler, Craig P. Hersh, Hemant K. Tiwari, Mark Dransfield, Anna Thalacker‐Mercer, Deborah A. Meyers, Edwin K. Silverman, Merry‐Lynn N. McDonald & on behalf of the COPDGene, ECLIPSE and SPIROMICS investigators (2021), ‘Genetic variation in genes regulating skeletal muscle regeneration and tissue remodelling associated with weight loss in chronic obstructive pulmonary disease’ (https://onlinelibrary.wiley.com/doi/10.1002/jcsm.12782), *Journal of Cachexia, Sarcopenia and Muscle*, Volume 12, Issue 6, 23 of the participants in the study (*n* = 4,000) did not consent to have their genetic data analysed, and should not be included in the study sample. The correct sample size should be 3,977. The authors wish to highlight this and confirm that removing them from the study sample does not affect the overall conclusion of the article.

